# Efficacy of an oral hygiene intervention on a geriatric ward: from sample size calculation to dropout-driven disillusionment - a randomised controlled study

**DOI:** 10.1186/s12903-025-07047-2

**Published:** 2025-10-21

**Authors:** Nora Marie Eckhardt, Max von Kohout, Dirk Bleiel, M. Cristina Polidori, Anna Greta Barbe

**Affiliations:** 1 University of Cologne, Faculty of Medicine and University Hospital Cologne, Polyclinic for Operative Dentistry and Periodontology, Cologne, Germany; 2 Institute of Medical Statistics and Computational Biology, Faculty of Medicine, University Hospital of Cologne, Cologne, Germany; 3 Ageing Clinical Research, Department II of Internal Medicine and Centre for Molecular Medicine Cologne, Faculty of Medicine and University Hospital Cologne, Cologne, Germany; 4 CECAD Cluster of Excellence for Aging Research, University of Cologne, Cologne, Germany

**Keywords:** Dental care for aged, Geriatric dentistry, Oral health, Preventive dentistry

## Abstract

**Background:**

Older home-dwelling adults with care needs often demonstrate deficient oral hygiene due to cognitive and motor impairments, as evidenced by increased plaque levels, which can compromise oral and general health. Concurrently, dental care utilisation declines with age. Hospitalisation may represent a critical opportunity to address this gap. However, dental assessments are not routinely integrated into multiprofessional inpatient geriatric care. Therefore, the aim of this study was to evaluate the efficacy of an oral hygiene education intervention on the plaque index compared with standard of care (SoC) among older frail acutely ill hospitalised inpatients.

**Methods:**

In this randomised controlled trial (RCT), hospitalised older inpatients were allocated to an intervention or control group. The intervention group participated in a structured oral hygiene education program including theoretical instruction on oral health and practical assistance in developing a personalised oral hygiene routine during their hospital stay. The control group received standard of care (SoC) on ward without additional oral hygiene support. Dental assessments were conducted at baseline (BL), pre-discharge (FU-1), and three months post-discharge (FU-2). Data on geriatric syndromes, comprehensive geriatric assessment (CGA) and the CGA-based Multidimensional Prognostic Index (MPI) were collected. The oral hygiene intervention and all assessments were conducted by the study dentist.

**Results:**

Among 36 participants (mean age 79.6 ± 8.1 years), 16 participants of the intervention group (*n* = 19) achieved a significant reduction in plaque index according to Silness Loe (PI; range 0–3) between BL (mean 2.4 ± 0.5) and FU-1, with an average improvement of 0.5 points (*p* < 0.001, *d* = 1.2), while controls (*n* = 17) showed no improvements (*p* > 0.05; *d* = − 0.1). Higher oral health-related quality of life (OHRQoL) scores were linked to better outcomes, while immobility resulted in reduced improvement in oral hygiene. Follow-up at FU-2 was limited by health-related attrition, with only four participants completing the study.

**Conclusions:**

Hospital-based diagnostics revealed widespread oral health problems of dependent home-dwelling individuals. The intervention improved oral hygiene in this high-risk group during hospitalisation. Integrating oral health diagnostics and interventions into geriatric care plans could address this critical aspect of patient health.

**Trial registration:**

The study is registered at German Clinical Trials Register (DRKS00027438; date of registration: February 13, 2023).

## Background

 Modern geriatric medicine emphasizes a multi-professional approach to addressing the complex needs of patients of advanced age with multimorbidity. The comprehensive geriatric assessment (CGA) encompasses age-related chronic illnesses, functional deficits, cognitive impairments, mental health challenges, nutritional status and social determinants of health, leading to the development of individualised care plans delivered by a multidisciplinary team [1]. This holistic approach is vital, given the high prevalence of functional limitations and cognitive impairments in older adults, which often compromise their capacity for self-care [2–4]. Oral health is a critical yet frequently overlooked aspect of geriatric care [3, 5]. Poor oral health and hygiene are prevalent in frail and multimorbid older adults and are associated with adverse systemic outcomes such as malnutrition, sarcopenia, dysphagia, aspiration pneumonia, and diminished quality of life [6–10]. Research by Noetzel et al. (2020) highlights the prognostic significance of oral health in older adults, demonstrating its correlation with the CGA-based Multidimensional Prognostic Index (MPI) [11] which is a validated tool for mortality prognosis [12].

Despite the importance of oral health, data from the Fifth German Oral Health Study (DMS V) reveal a decline in control-oriented dental care utilisation with advancing age and increased care needs. While 76% of patients with excellent general health seek preventive dental care, this figure drops to 38% among those with poor general health. Similarly, utilisation of dental care declines from 90% in the 65–74 age group to 62% in those aged 75–100 [13].

For individuals of advanced age living independently or with family assistance, physical and logistical barriers often limit access to dental services [14]. Acute health events requiring hospitalisation in geriatric units offer a unique opportunity to address this gap. However, standardised dental examinations are not routinely included in hospital-based care, despite their potential to facilitate oral health interventions [15, 16]. Integrating dental care into the multi-professional framework of acute geriatric wards—not only in terms of screening and diagnostics, but also increasing awareness about oral hygiene issues—could address unmet oral health needs and potentially improve long-term outcomes and has not yet been explored [17].

A previous randomised controlled trial by Croonquist et al. (2020) demonstrated that implementing a monthly regimen of individualised oral hygiene education and professional cleaning by dental hygienists led to reduced plaque scores and a lower incidence of gingival bleeding among nursing home residents [18]. In light of the frequent neglect of oral hygiene in hospital settings, as evidenced by elevated plaque scores [19, 20], our aim was to evaluate whether providing personalised oral hygiene education during a patient’s stay could improve their oral health and hygiene, both during their stay and after discharge, compared to the standard of care (SoC) group. Specifically, our primary outcome was defined as a one-point improvement in each participant’s individual plaque index (PI) between baseline (BL) and three months after discharge (FU-2).

## Methods

### Ethics and study registration

The study received approval from the Ethics Committee of the Medical Faculty at the University of Cologne (approval number: 21-1486_2; date: January 30, 2023), and was registered in the German Clinical Trial Register (DRKS; registration number: DRKS00027438; date of registration: February 13, 2023). The study adhered to the ethical principles outlined in the Declaration of Helsinki, and to the guidelines for Good Clinical Practice, as revised on January 17, 1997.

### Study design

The study was designed as a longitudinal prospective, randomised controlled trial (RCT) with an intervention and control group. Participants were allocated to either group in a 1:1 ratio. The intervention group participated in a structured oral hygiene education program while the control group received SoC.

### Sample size

The dental plaque index (PI) according to Silness and Loe [21] (scale 0–3) is a well-established metric for assessing oral hygiene in geriatric patients and reflects the quality of oral care, which is often compromised by age-related motor and cognitive impairments [17, 22]. Our primary outcome was a one-point improvement in the individual PI per participant between baseline and follow-up 2 (FU-2). Previous research by Barbe et al. (2019) on institutionalised patients of advanced age with an oral hygiene intervention demonstrated a one-point PI improvement in 30% of participants in the intervention group, compared to no change in the control group [23]. Based on these findings, the required sample size was calculated to detect a one-point difference in PI scores between the intervention and control groups with 80% power and a significance level of 0.05. The calculation determined that 24 participants per group were necessary. To account for an anticipated 25% dropout rate, the sample size was adjusted to 32 participants per group, resulting in a total of 64 participants. To minimize dropout, a free professional dental cleaning was offered to all participants at the final follow-up appointment (FU-2).

### Participants

Participants were enrolled in the study based on the following inclusion criteria: (a) residing at home; (b) aged 60 years or older; (c) multimorbid, defined as having more than two illnesses requiring treatment; (d) possessing at least four natural teeth; (e) PI score ≥ 1; and (f) provision of informed consent by the patient or their legal guardian. Exclusion criteria included: (a) insufficient proficiency in German to complete the questionnaires; (b) a diagnosis of dementia confirmed by a neurologist; (c) life-threatening organ failure with an expected fatal outcome during the study period; and (d) the requirement for antibiotic prophylaxis for dental examinations, such as for endocarditis prevention. Participants were recruited from inpatients at the University Hospital of Cologne, Department of Geriatric Medicine, Clinic II for Internal Medicine - Nephrology, Rheumatology, Diabetology, and General Internal Medicine, in Germany. Eligible patients were those receiving early geriatric complex rehabilitation which usually lasts for a duration of 14 days.

### Early geriatric complex rehabilitation (EGCR)

Early geriatric complex rehabilitation (EGCR), as defined by the Operation and Procedure Code in Germany, involves care provided by a multi-disciplinary geriatric team [24]. This approach includes activating therapeutic care and covers several therapeutic areas: physiotherapy, occupational therapy, speech therapy, and (neuro)psychology. At both the beginning and end of treatment, a CGA is conducted, evaluating mobility, self-care abilities, cognition, and emotions. Additionally, a social assessment is performed, considering the individual’s social environment, living conditions, daily activities, care or assistance needs, and legal requirements. Dental expertise from a dentist or dental specialist is not currently a mandatory component of EGCR [24].

### Study procedures

All patients who met the eligibility criteria were enrolled in the study over a one-year period at the beginning of their hospital stay (March 2023 to March 2024). The last follow-up was concluded in April 2024. All assessments, randomisation and the intervention were conducted by the same dentist (N.M.E).

#### Baseline and randomisation

At baseline, oral health assessment and CGA were performed. These included the collection of the PI and the MPI. Based on the PI score, participants were classified into one of two groups: PI1 (mean PI 1 to < 2) and PI2 (mean PI 2 to 3). They were also assigned to one of three MPI groups: MPI1 (0.00–0.33.00.33), MPI2 (0.34–0.66), or MPI3 (0.67–1.00.67.00). The randomisation process was conducted within the ward, stratified by MPI and PI classes (MPI1, MPI2, MPI3, and PI1, PI2). This involved using prepared, sealed, and sequentially numbered randomisation envelopes in six strata. The following groups were created: (MPI1-PI1-XX, MPI1-PI2-XX, MPI2-PI1-XX, MPI2-PI2-XX, MPI3-PI1-XX, MPI3-PI2-XX). Participants were randomised into either the intervention or control group. The randomisation letters were prepared at the Institute of Medical Statistics and Computational Biology, Faculty of Medicine and University Hospital of Cologne.

#### Oral hygiene intervention group

**Fig. 1:**
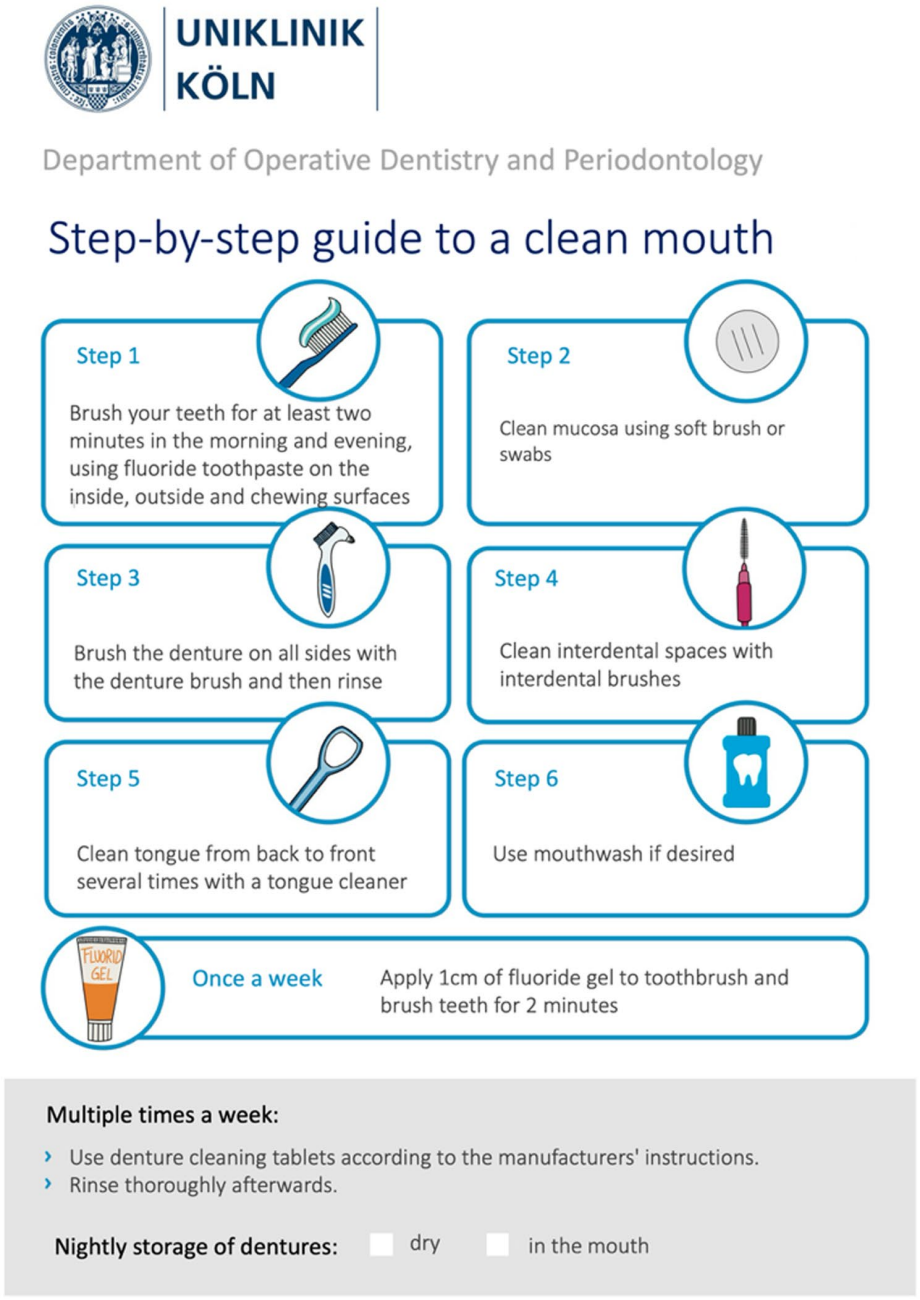
Oral hygiene flyer (translated version from german)

Sixteen participants in the intervention group received oral hygiene training at the beginning of their hospital stay, which included both theoretical education on oral health and practical instruction on professional tooth brushing. The training emphasised the importance of maintaining good oral hygiene for overall health, with a specific focus on preventing aspiration pneumonia and preserving teeth to ensure adequate chewing ability. The goal of the instruction was to optimise existing hygiene practices and, if necessary, introduce new aids. The German Network for Quality Development in Nursing (DNQP) expert standard “Promoting Oral Health In Nursing” [25] provided the evidence-based foundation for the oral hygiene routine. Any missing aids needed to perform the routine were provided to the participants. A structured oral hygiene routine was outlined using a flyer (Fig. 1), and the brushing technique was demonstrated using a typodont. The visiting time was approximately 30 min.

##### Tooth brushing

Particular emphasis was placed on a systematic approach to cleaning all tooth surfaces. If motor skills were sufficient, participants brushing their teeth with a manual toothbrush were instructed to use the modified Bass technique; if not, the Fones technique was used [26]. For participants using an electric toothbrush, special attention was given to ensure all tooth surfaces were thoroughly cleaned. All participants were provided with fluoride toothpaste (1.400 ppm fluoride, Parodontax Gum Health Clean Mint Toothpaste; Haleon, London, UK). Daily cleaning of interdental surfaces with appropriately sized interdental brushes was recommended for all participants.

##### Tongue cleaning

Participants with a high tongue coating index [27] (>5) were advised to clean their tongue daily with a tongue cleaner.

##### **Denture hygiene**

Dentures that had tartar were cleaned once in an ultrasonic bath (Cleaning fluid Stammopur Z, Bandelin, Berlin, Germany) by the study dentist. As described in the DNQP expert standard daily cleaning of dentures with a denture brush and toothpaste was recommended, along with disinfection twice a week in a denture cleaning bath (Kukident Aktiv Clean; Reckitt Benckiser, Heidelberg, Germany). Participants were instructed to store their dentures overnight in a dry place, if tolerated – that is, only if removing the dentures during the night does not cause significant discomfort or distress – and to remove food residues or denture adhesive cream from the oral mucosa every day with a cotton swab [28].

##### Implementation of the routine

After receiving instructions, participants performed the recommended oral hygiene routine as outlined in the flyer to the best of their ability, under the supervision of the study dentist. The brushing was then re-evaluated and, if necessary, the dentist professionally brushed the participants’ teeth using the toothbrush and interdental brushes until no visible plaque remained. Participants were then asked to rate their motivation to implement a new oral hygiene routine on a scale from 0 to 10 (0 indicating no motivation to improve oral hygiene; 10 indicating high motivation to adopt the new routine). One week later, their oral hygiene was reassessed and, if needed, additional practice was provided.

#### Control group

Participants in the control group received SoC in the ward. Although this encompassed needs-based assistance with personal hygiene by nursing staff, it did not include standardised oral hygiene support. Furthermore, the participants of the control group did not receive any oral hygiene education intervention or practice from the study dentist.

#### Follow-up 1

Follow-up 1 (FU-1) was conducted one day before discharge. The oral health assessment was repeated for all participants.

#### Follow-up 2

Three months after discharge, all participants were invited to attend FU-2 at the dental clinic. During this appointment, the oral health assessment and MPI were conducted again.

### Parameters assessed

#### Geriatric outcomes

For the geriatric outcomes, the CGA, the CGA-based MPI [11] (representing mortality prognosis) which consists of eight domains including the number of medications and the Cumulative Illness Rating Score (CIRS) [29] were recorded. The sum of the calculated scores is divided by eight to generate the final MPI score, which ranges from 0.00 (lowest mortality risk) to 1.00 (highest mortality risk). Appropriate validated cut-offs have been calculated to identify three levels of mortality risk as follows MPI-1, 0–0.33.33 = low risk, MPI-2, 0.34–0.66 = moderate risk, and MPI-3, 0.67–1.67, high risk [11]. The CIRS provides information about the number of medical conditions requiring long-term treatment. Furthermore, the duration of stay, geriatric syndromes [30] (which capture age-associated clinical conditions), the Barthel Index [31] (BI; a comprehensive assessment of a range of activities of daily living including self-care), the Self Care Index [32] (SPI; score including 10-items used to predict post-acute care deficits and to serve as an indicator for the severity of nursing dependency) as part of the CGA were recorded.

#### Oral health parameters

At baseline and FU-1, the dental examinations were performed at the bedside of participants. At FU-2, the dental examination was performed in the Polyclinic for Operative Dentistry and Periodontology, University of Cologne, Cologne, Germany. The following parameters were recorded at each study appointment: number of teeth, PI according to Silness and Loe [33] (assessed at two sites per tooth, oral and buccal; range from 0 to 3), Decayed, Missing or Filled Teeth Index (DMFT) [34], Periodontal Screening Index [35] (the German adaptation of the Community Periodontal Index of Treatment Needs (CPITN) [36] used to document signs of gingivitis and periodontitis, allowing differentiation between moderate (3.5–5.5 mm) and deep (≥ 5.5 mm) periodontal pockets and indicating potential need for periodontal treatment), Papilla Bleeding Index [37], Root Caries Index according to Katz [38], Tongue Coating Index according to Winkel [27], Denture Hygiene Index according to Wefers [39], Oral Health Literacy Profile (OHLP) which assesses oral hygiene behaviour (e.g. toothbrushing frequency, use of oral hygiene products) and oral-health related self-perception [40], and Geriatric Oral Health Assessment Index (GOHAI) [41]. In order to evaluate oral health related self-efficacy we asked participants one question as administered by the Social And Dental Health Science Questionnaire of the DMS V [13]. Participants of the intervention group were asked to rate their motivation to implement the new oral hygiene routine on a scale from 0 to 10 (0 indicating no motivation to improve oral hygiene; 10 indicating high motivation to adopt the new routine) after receiving the intervention.

### Statistical analysis

The data were analysed using IBM SPSS Statistics (version 22.0, IBM Corporation, New York, NY, USA) and R (Version 4.3.1, R Foundation for Statistical Computing, Vienna, Austria), with statistical significance set at 0.05. The mean and standard deviation (±) were provided for the quantitative variables. Parametric (e.g., t-test) and non-parametric (e.g., Mann-Whitney U-test, Eta-correlation) tests were applied based on variable distribution. Spearman and Pearson correlations were used to assess relationships between continuous variables. Due to the high drop-out rates at FU-2, the clinical effect of the intervention was determined by the differences in change in PI from baseline to FU-1. Primarily, the outcome was investigated by a pairwise t-test adjusting for unequal group sizes and variances. Furthermore, a multiple linear regression model using ordinary least squares estimation was utilised to explore the effect of intervention and identify relevant covariates and their indirect effects. The most plausible and robust model was selected through a sensitivity analysis as backward selection algorithm (Appendix 1).

Finally, the estimated marginal means of the model were calculated to investigate the intervention effect on population subgroups via pairwise comparisons with Tuckey correction for multiple testing.

## Results

**Fig. 2:**
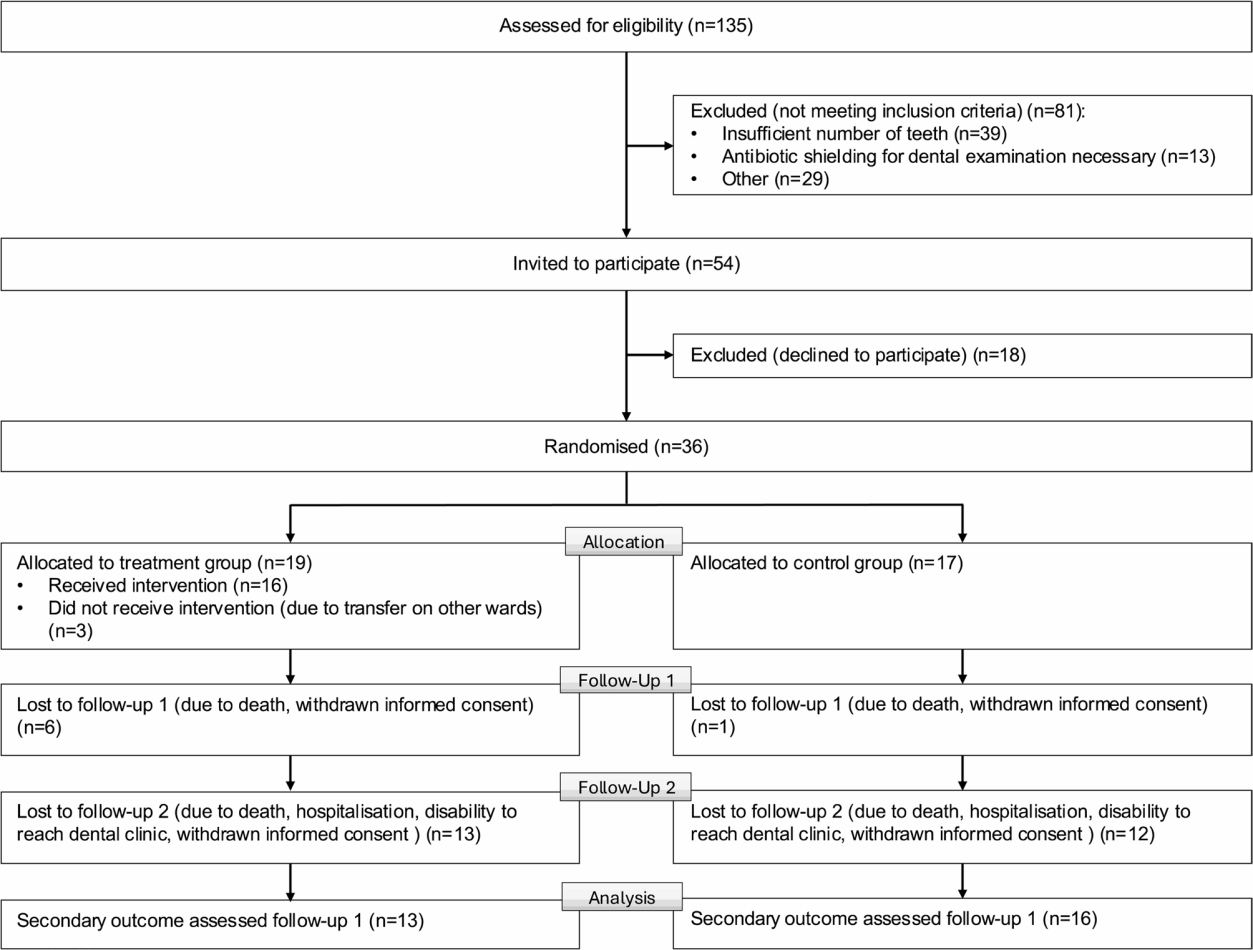
CONSORT study flowchart

The CONSORT flowchart with participant numbers is shown in Fig. 2. A total of 135 patients were screened, of whom 81 did not meet the eligibility criteria. The majority of exclusions were due to an insufficient number of teeth (*n* = 39), the need for antibiotic prophylaxis during the dental examination (*n* = 13), or other exclusion criteria (*n* = 29). Fifty-four patients were invited to participate in the study, and 36 patients or their legal guardians provided informed consent, resulting in a participation rate of 26.7% among all screened patients. Participants were randomised into the intervention group (*n* = 19) or the control group (*n* = 17). However, the 1:1 randomisation was not achieved due to the sample size not being reached. Three participants in the intervention group dropped out before receiving the intervention due to transfer to other wards. In total, the dropout rate was 19.4% (*n* = 7) at FU-1, primarily due to medical reasons (*n* = 4) or death (*n* = 3), which increased to 88.9% at FU-2. This left 29 participants completing the trial until FU-1 and only four participants completing the 3-month follow-up (FU-2). Screening was concluded after one year.

### Participant and clinical characteristics at baseline

Table 1 shows the baseline demographic and clinical characteristics of participants in the intervention and control groups. The mean age of participants was 79.6 ± 8.1 years (range 64–94 years), and 63.9% were male. There were no significant differences in oral and geriatric characteristics, except for the number of teeth (*p* = 0.021).

**Table 1:** Clinical characteristics of the study population at baseline

	Acute geriatric inpatients			*p*-value *
	Total (*N* = 36) ^*a*^	Intervention (*n* = 19) ^*a*^	Controls (*n* = 17) ^*a*^	
Age (years)	79.6 ± 8.1 (64–94)	78.7 ± 7.9 (64–91)	80.6 ± 8.4 (67–94)	0.500
MPI	0.53 ± 0.15 (0.31–0.81)	0.55 ± 0.15 (0.31–0.81)	0.52 ± 0.14 (0.31–0.81)	0.565
CIRS	5.9 ± 1.9 (3–9)	5.8 ± 2.0 (3–9)	6.0 ± 1.8 (3–8)	0.748
Number of medications	10.0 ± 3.7 (4–19)	9.5 ± 3.8 (4–17)	10.7 ± 3.6 (6–19)	0.352
Duration of stay in days	21.6 ± 13.1 (1–60)	21.8 ± 15.4 (1–60)	21.4 ± 10.4 (9–46)	0.923
BI	44.2 ± 18.2 (10–85)	40.5 ± 15.4 (10–65)	48.2 ± 20.6 (10–85)	0.209
Sum of geriatric syndromes	6.0 ± 2.4 (1–11)	6.0 ± 2.4 (1–10)	6.1 ± 2.5 (2–11)	0.943
Number of teeth	16.2 ± 7.4 (5–29)	13.6 ± 7.2 (5–29)	19.2 ± 6.6 (6–29)	**0.021**
DMFT Index	22.1 ± 4.3 (14–28)	21.4 ± 4.6 (14–28)	22.8 ± 4.1 (15–28)	0.360
PI	2.4 ± 0.5 (1.1–3.0.1.0)	2.4 ± 0.6 (1.1–3.0.1.0)	2.3 ± 0.5 (1.1–2.9)	0.580
Maximum PSI	3.2 ± 0.7 (1–4)	3.1 ± 0.8 (1–4)	3.3 ± 0.7 (1–4)	0.333
Maximum Degree of tooth mobility	1.0 ± 0.9 (0.0–2.0)	1.0 ± 0.9 (0–2)	0.9 ± 1.0 (0–2)	0.863
PBI	1.2 ± 0.9 (0.0–3.3.0.3)	1.4 ± 0.9 (0.14–3.3)	0.9 ± 0.7 (0.0–2.5.0.5)	0.094
RCI	0.3 ± 0.3 (0–1)	0.3 ± 0.3 (0–1)	0.3 ± 0.2 (0.0–0.3.0.3)	0.636
Tongue Coating Index	6.1 ± 4.7 (0.0–12.0)	5.4 ± 4.8 (0.0–12.0)	6.9 ± 4.6 (0.0–12.0)	0.344
DHI, Upper Prothesis	6.7 ± 3.1 (0.0–10.0) (*n* = 19)	6.8 ± 2.9 (0.0–10.0) (*n* = 13)	6.5 ± 3.7 (0.0–10.0) (*n* = 6)	0.821
DHI, Lower Prothesis	7.6 ± 3.8 (0.0–10.0) (*n* = 12)	7.2 ± 4.2 (0.0–10.0) (*n* = 9)	9.0 ± 1.7 (0.0–10.0) (*n* = 3)	0.679
Total GOHAI Score	48.0 ± 7.5 (34–60)	46.4 ± 7.8 (34–58)	49.7 ± 6.9 (37–60)	0.191

*Abbreviations: BI* Barthel Index, *CIRS* Cumulative Illness Rating Score, *DMFT* Decayed Missing Filled Teeth Index, *MPI* Multidimensional Prognostic Index, *PBI* Papillary bleeding index, *PI* Plaque Index (Silness Loe), *PSI* Periodontal Screening Index, *RCI* Root Caries Index, *DHI* Denture Hygiene Index (Wefers), *GOHAI* Geriatric Oral Health Assessment Index

Results presented as mean ± standard deviation (range)

* Two-sided T-test or Mann-Whitney U test based on variable distribution; p value < 0.05 considered significant (bold)

^*a*^ Unless otherwise indicated

### Geriatric characteristics

All participants exhibited severe health conditions, with a mean of 5.9 ± 2 illnesses that required medical or surgical intervention. The median length of hospitalisation was 21.6 ± 13.1 days. The mean MPI at the time of admission was 0.53 ± 0.15, with 8.3% of participants (*n* = 3) assigned to MPI-1, 69.4% (*n* = 25) to MPI-2, and 22.2% (*n* = 8) to MPI-3. The mean BI at admission was 44.3 ± 18.2. Participants had a mean of 6 ± 2.4 geriatric syndromes, with polypharmacy (*n* = 32), instability (*n* = 30), and impaired hydration status (*n* = 21) being the most prevalent.

### Oral health-related characteristics

Participants had a mean of 16.2 ± 7.4 natural teeth and a mean DMFT index value of 22.1 ± 4.3. All participants exhibited elevated plaque levels (mean PI 2.4 ± 0.5) and signs of gingivitis. Thirty-one subjects (86.1%) exhibited moderate to deep periodontal pockets, suggesting a necessity for periodontal treatment. The mean Tongue Coating Index was 6.1 ± 4.7 (range 0.0–12.0) while the mean Denture Hygiene Index scores were 6.7 ± 3.1 (range 0.0–10.0) for upper dentures and 7.6 ± 3.8 (range 0.0–10.0) for lower dentures. However, 23 participants (64%) believed their dental status to be satisfactory to excellent. Twenty-eight participants (77.8%) reported using a manual toothbrush, while eight (22.2%) used an electric toothbrush. Notably, none of the participants brought an electric toothbrush with them to the ward. Participants had a mean total GOHAI score of 48.0 ± 7.5.

### Motivation to improve oral hygiene

The mean motivation to perform the optimised oral hygiene routine among participants in the intervention group was 6.36 ± 3.0 (range 1–10), indicating a mean moderate to high level of motivation.

### Correlation between oral health and geriatric parameters

Self-assessments of dental status (η² = 0.208), toothbrushing frequency (η² = 0.304) and brushing duration (η² = 0.304) showed no correlation with clinical oral health, suggesting that the perceptions of participants did not accurately reflect their actual oral hygiene.

### Characterisation of excluded participants

Table 2 presents the baseline demographics and clinical characteristics of enrolled participants and excluded participants. No significant differences were found between included participants and excluded participants in the clinical characteristics examined, except for the number of medications and the Self Care Index (SPI).

**Table 2:** Clinical characteristics of excluded participants and enrolled participants at baseline

	Acute geriatric inpatients			Test	*p*-value	Effect size *r*
	Total (*N* = 135) ^*a*^	Study Population (*n* = 36) ^*a*^	Excluded Participants (*n* = 99) ^*a*^			
Age (years)	79.7 ± 8.8 (54–98)	79.6 ± 8.1 (64–94)	79.7 ± 9.1 (54–98)	t-Test	0.964	
Duration of stay (days)	21.4 ± 18.5 (1–183)	21.6 ± 13.1 (1–60)	21.3 ± 20.2 (1–183)	Mann-Whitney U-Test	0.519	
CIRS	6.2 ± 2.1 (3–15)	5.9 ± 1.9 (3–9)	6.3 ± 2.3 (3–15)	Mann-Whitney U-Test	0.556	
Sum of geriatric syndromes	6.2 ± 2.3 (1–15) (*n* = 122)	6.0 ± 2.4 (1–11)	6.3 ± 2.3 (2–15) (*n* = 86)	Mann-Whitney U-Test	0.69	
Number of medications	11.3 ± 4.5 (2–29)	10.0 ± 3.7 (4–19)	11.8 ± 4.7 (2–29)	Mann-Whitney U-Test	0.046*	0.17
BI	40.5 ± 19.1 (0–95) (*n* = 131)	44.2 ± 18.2 (10–85)	39.11 ± 19.4 (0–95) (*n* = 95)	t-Test	0.176	
Selfcare Index	28.0 ± 6.8 (10–40) (*n* = 129)	30.2 ± 5.5 (15–39)	27.2 ± 7.1 (10–40) (*n* = 93)	Mann-Whitney U-Test	0.044*	0.18

*Abbreviations: BI* Barthel Index, *CIRS* Cumulative Illness Rating Scale

Results are presented as mean ± standard deviation (range)

^*a*^ Unless otherwise indicated

* *p* value < 0.05 considered significant (bold)

### Intervention results and regression modelling

Table3 shows the effects in PI for intervention and control group. The intervention group demonstrated a significant improvement in PI, with a decrease from 2.4 ± 0.6 at baseline to 2.0 ± 0.7 at FU-1 (*p* < 0.001; *d* = 1.2). In contrast, the control group exhibited no improvement (*p* > 0.05; *d*=−0.1). No participant showed PI-improvement by one-point at FU-1

**Table 3:** Development of the PI in intervention and control group at baseline and FU-1

	Intervention Group	Control Group
PI, BL	2.44 ± 0.58 (1.1–3.0.1.0) (*n* = 19)	2.34 ± 0.5 (1.1–2.9) (*n* = 17)
PI, FU-1	2.0 ± 0.7 (0.5–2.9) (*n* = 13)	2.4 ± 0.4 (1.4–2.9) (*n* = 16)
p-value*	< 0.001***	0.301

*Abbreviations: PI,* Plaque Index (according to Silness Loe), *BL* Baseline, *FU-1* Follow-up 1

Results presented as mean ± standard deviation (range)

*Single sided T-test

*p-value < 0.05 considered significant

***p-value < 0.001 considered highly significant

**Fig. 3:**
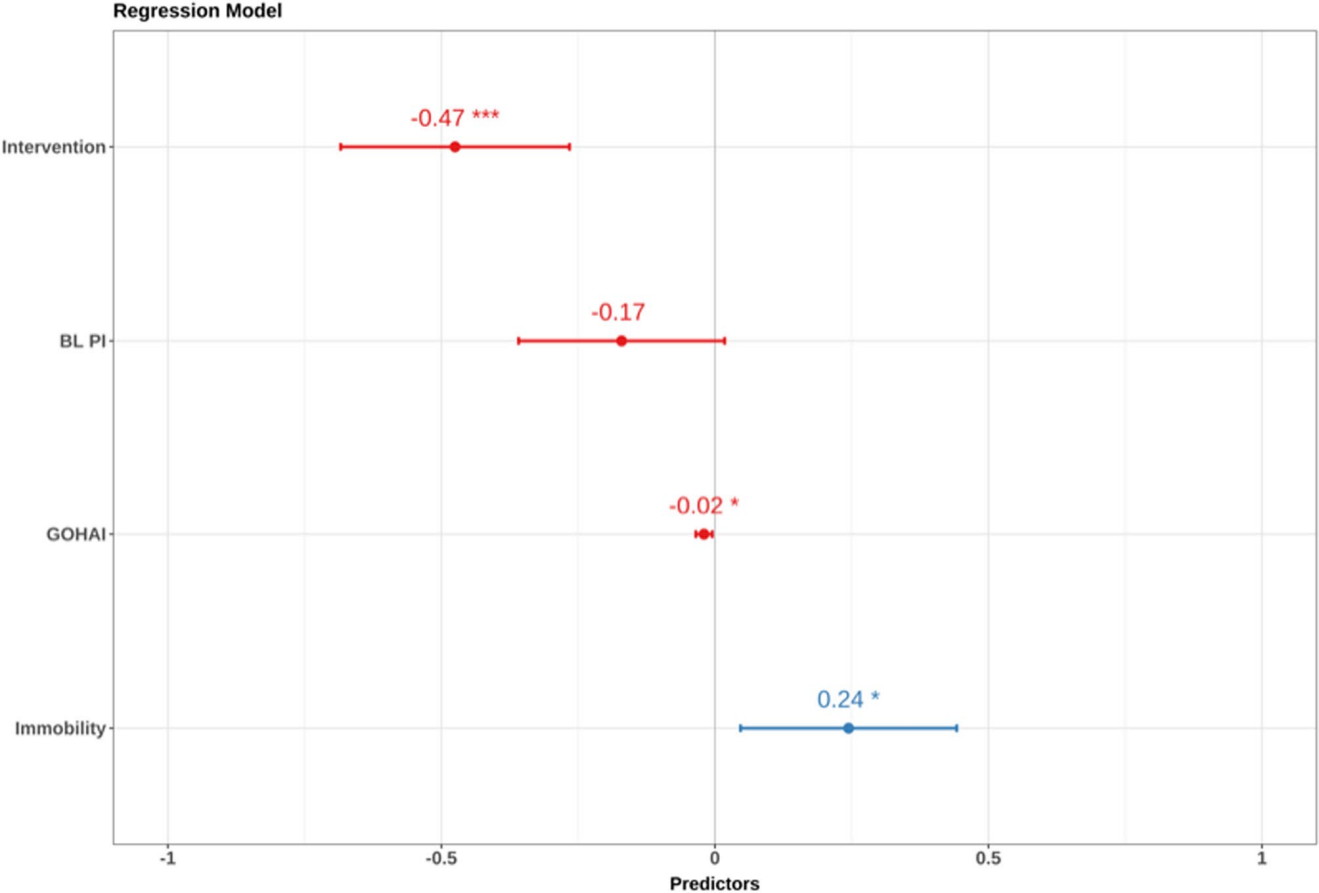
Multiple linear regression model including four predictors: intervention, geriatric oral health assessment index (GOHAI), and immobility. Notes: *p-value < 0.05 considered significant; *** p-value < 0.001 considered highly significant. Abbreviations: GOHAI, Geriatric Oral Health Assessment Index

For the model selection procedure, a parsimonious model was chosen, which was gradually adapted by variables chosen on clinical expertise, the stepwise selection algorithm, and the investigated correlation matrix. The final model included four predictors: intervention, GOHAI, and immobility, demonstrating good fit (adj. R² = 0.56, *p* = 0.001), explaining 56% of the variance in PI change. The intervention resulted in an improvement in PI of 0.5 units (Fig. 3). Alternative predictors (e.g., age, gender, MPI, depression, number of teeth, geriatric syndromes) showed no significant effects.

To assess the robustness of our regression findings, we employed bootstrap resampling with 5,000 replications to generate alternative confidence intervals and p-values. The results from these robust estimation procedures were consistent with the original ordinary least squares (OLS) analysis, showing no changes in coefficient signs or statistical significance levels.

Regression assumptions were validated via visual inspection and diagnostic tests (Appendix 2).

**Fig. 4:**
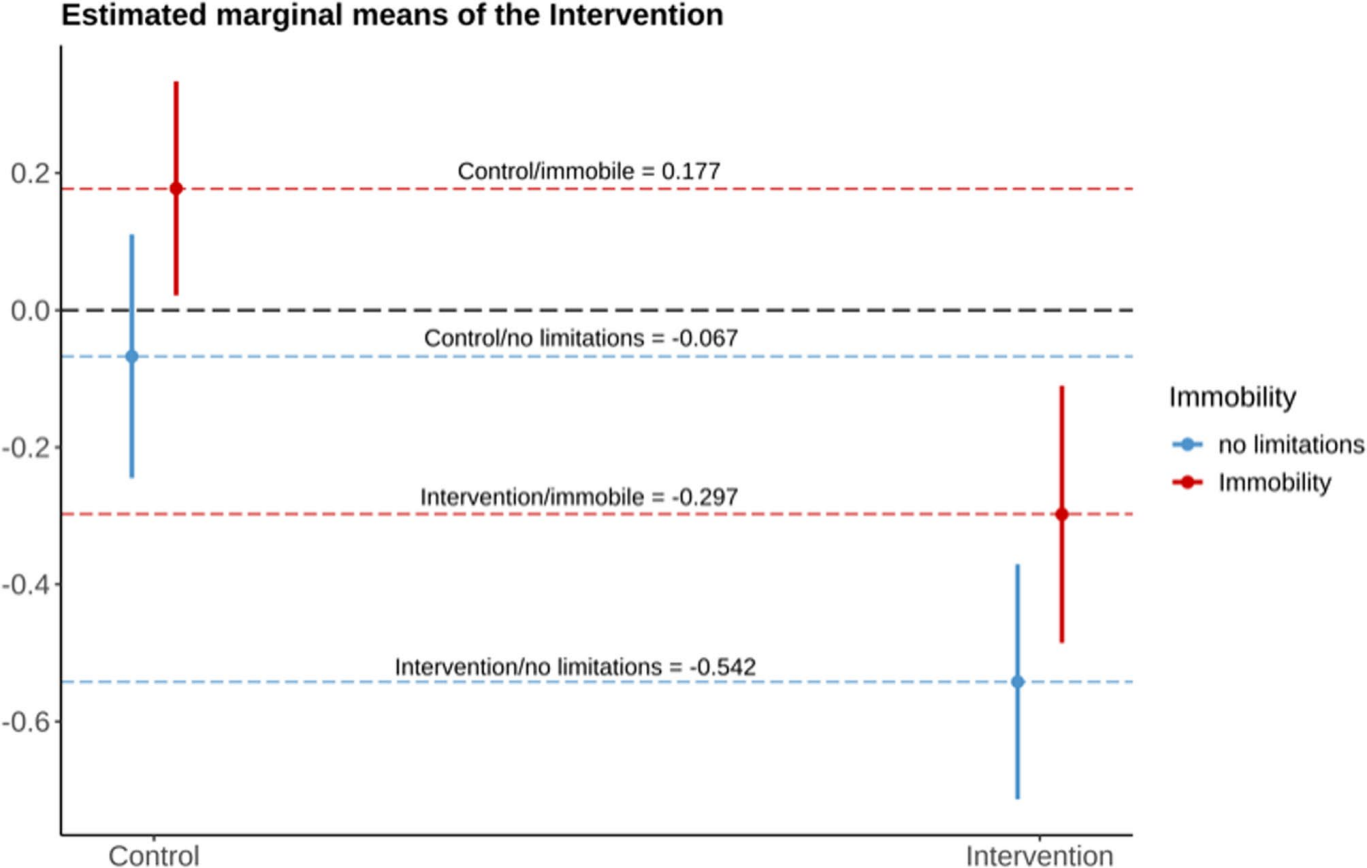
Estimated marginal mean differences for the combination of intervention and immobility. Adjusted for Plaque index at baseline (BL PI) and geriatric oral health assessment index (GOHAI)

Figure 4 presents the models estimated marginal means for the combination of intervention and immobility, adjusted for the population mean of PI (baseline) and GOHAI.

The analysis of population subgroups based on pairwise comparisons between fully mobile participants of the control and intervention group suggests a decrease in PI in the intervention group of 0.5 units (*p* < 0.0001). The final comparison revealed the potential of the intervention in mobile participants compared to immobile participants in the control group, with an improvement in PI of 0.7 units (*p* < 0.0001) (Table 4). In the control group, there was no change in PI between BL and FU-1 (*p* > 0.05).

**Table 4:** Models of estimated marginal means regarding immobility

Pairwise Comparisons	Estimate	SE	t-ratio	*p*-value
i) Intervention (no limitations) (*n* = 6) vs. Control (no limitations) (*n* = 7)	0.5	1.010	4.686	< 0.001***
ii) Control (no limitations) (*n* = 7) vs. Control (immobility) (*n* = 9)	0.2	958	−2.554	**0.08†**
iii) Control (no limitations) (*n* = 7) vs. Intervention (immobility) (*n* = 7)	0.2	1.500	1.535	0.48
iv) Intervention (no limitations) (*n* = 6) vs. Control (immobility) (*n* = 9)	0.7	1.280	−5.628	< 0.001***

*Abbreviations: SE *standard error

Tuckey corrected for multiple testing

p-value < 0.05 considered significant (bold)

†, *p* < 0.1 >0.05

*** *p* < 0.001

## Discussion

 We explored the impact of an educational oral hygiene intervention on a geriatric ward. To our knowledge, this is one of the first studies to implement an oral hygiene education program specifically in an acute geriatric setting, targeting patients who were residing in their own homes at the time of hospitalisation. The single, brief intervention led to improvements in oral hygiene parameters during the period of hospitalisation. However, the primary endpoint, which was the one-point improvement in the individual PI at FU-2 could not be assessed due to the high drop-out rate at FU-2 (Figure 2). Consequently, no conclusions regarding the long-term efficacy of the intervention can be drawn. It is noteworthy that the observed improvements during hospitalisation occurred despite the population being characterised by severe systemic health conditions, a high prevalence of geriatric syndromes, and elevated MPI at admission (Table 1). The consequent limited capacity to perform satisfactory oral hygiene was demonstrated by significant deficiencies in both oral health and hygiene at admission (Table 1). Even if the impact of the intervention was limited to the inpatient period, the short-term effect during hospitalisation, although modest, remains valuable. The bedridden state induced by illness significantly elevates the risk of aspiration pneumonia, and improving oral hygiene in this period could help mitigate that risk [3, 9]. Nonetheless, the intervention effect was inadequate in all participants to ensure adequate oral hygiene on its own, emphasising the necessity for further and ongoing measures to promote oral hygiene that extend beyond the acute inpatient setting. Our results highlight the value of utilising acute geriatric settings as an interface for dental assessments, which can uncover oral health issues and guide necessary treatments. The complex aetiology of poor oral health necessitates multifaceted, coordinated interventions to achieve lasting improvements.

One of the key factors influencing changes in oral hygiene was the GOHAI total score at admission. A higher GOHAI score was associated with greater improvements in plaque levels, suggesting a relationship between the particpants’s oral health-related quality of life and the effectiveness of the intervention. However, Nordblom et al. (2024) demonstrated in a recent study that the remotivation and re-instruction of nurses to implement oral hygiene routines in moderate to high care dependent nursing home residents did not improve oral health-related quality of life [42]. Participants with better self-perceived oral health appeared to be more likely to benefit from the intervention, reinforcing the idea that a positive self-assessment of oral health may drive better oral hygiene outcomes. This aligns with findings from Reisine et al. (2021), who demonstrated that an oral hygiene intervention using adapted motivational interviewing and brushing demonstrations could increase GOHAI scores [43]. Additionally, Allen et al. (2022) found that a higher self-perception of oral health correlated with better oral hygiene [44].

While many studies have explored the link between oral health and conditions such as dysphagia, xerostomia, sarcopenia, and frailty, the relationship between immobility and oral hygiene remains underexplored [8, 45, 46]. Our findings suggest that immobility reduces improvement in oral hygiene outcomes. Immobile patients often face significant challenges in accessing toileting facilities, which may hinder their ability to maintain good oral hygiene. Furthermore, immobility has been demonstrated to be associated with a diminished health-related quality of life, in addition to pain and discomfort [47]. These factors may have also contributed to a deterioration in oral hygiene. These challenges highlight the need for tailored interventions that address the specific needs of immobile patients, ensuring that their oral health is adequately supported despite their physical limitations.

Given that the participants in this study resided at home, the involvement of their supportive environment in daily oral hygiene practices is critical. A significant proportion of participants in our study lacked access to outpatient care for assistance with oral hygiene routines, underscoring the importance of engaging family members or caregivers in this process. Research by Barbe et al. (2021) found that oral hygiene provided by trained laypersons can achieve plaque reduction levels comparable to those of dental professionals [48]. Relatives could receive support with oral hygiene from general dentists and trained dental assistants; for example, video consultations would make decentralised implementation possible and should be examined in further studies. Outpatient care services could also be included. This is particularly relevant for patients with limited ability to care for their oral hygiene at home. However, it is also essential to recognize that patient compliance may vary when oral hygiene care is provided by family members. Blasi et al. (2023) found that patients of advanced age often value autonomy in their oral care, which could influence their acceptance of assistance from family members [49]. Moreover, according to the Nordblom study, this suggests that, while participants requiring oral care support may benefit from laypeople's assistance in enhancing oral hygiene, this does not automatically translate into an improvement in oral health-related quality of life [42].

Conducting clinical research in this population presents numerous challenges. The inclusion of participants was particularly difficult due to the high prevalence of edentulism and poor systemic health among patients. Additionally, the standard geriatric assessments conducted upon admission were inadequate for identifying those most likely to benefit from the intervention. Many participants struggled to accurately assess their own oral hygiene, which may have contributed to their decreased understanding of the need for improvement. Furthermore, a significant portion of participants expressed that the daily oral hygiene routine felt burdensome, preferring to focus on oral care only when they felt better. In future studies, the inclusion of edentulous patients should be considered not only to increase recruitment rates, but also because inadequate denture hygiene has been associated with an elevated risk of aspiration pneumonia. In order to reduce the attrition rate and improve data completeness, it may be beneficial that further efforts be made to include family members and carers in order to improve coordination of access to study appointments and that follow-up assessments be conducted in participants’ homes if deemed necessary. Moreover, incorporating qualitative data (e.g. nursing or carer experience) would allow for the analysis of barriers hindering the implementation of oral hygiene routines.

### Limitations

The accuracy of the DMFT index in our study may have been compromised due to the presence of uncleaned tooth surfaces, which made effective caries detection difficult at baseline. Although the PI is a frequently utilised outcome measure for the evaluation of oral hygiene, it functions solely as a surrogate outcome, thereby lacking the capacity to directly assess critical clinical outcomes, including the development of caries or the progression of periodontal disease. A potential source of assessment bias in the study was that the study dentist was not blinded; however, to mitigate this, baseline measurements were not reviewed prior to the collection of follow-up data. In addition, oral hygiene progress could have been influenced by potential confounders such as oral care provided outside the intervention (e.g. by family members or nurses). Furthermore, the high dropout rate following FU-1 prevented us from reaching the predefined sample size, with only a small number of participants completing FU-2 which led to the inability to assess the primary endpoint and a high risk of attrition bias. Consequently, the study focused on the analysis between BL and FU-1. The reasons for dropout were primarily related to poor general health and the associated effort required to attend appointments. Many participants also perceived themselves as a burden on their relatives and were reluctant to request transportation to the dental department. These findings are consistent with challenges described in other studies of populations of advanced age [50]. Offering free dental cleaning did not significantly reduce the dropout rate, suggesting that oral health may be perceived as a lower priority among this population group, as indicated by Blasi et al. [49].

Even though the selected model showed strong evidence for the internal validity of the study, external validity is limited due to the relatively small number of cases as well as the single-centre and single-operator design of the study.

## Conclusion

We demonstrated that an educational oral hygiene intervention resulted in improvements in plaque index among multimorbid patients in an acute geriatric ward during hospitalisation. However, oral hygiene remained inadequate, and participants exhibited a lack of awareness regarding the need for oral hygiene support. The acute geriatric ward presents an important opportunity for dental clinicians to engage with patients who may not otherwise receive ongoing care. The implementation of structured oral health screening as a component of geriatric assessment has the potential to make a substantial contribution to the establishment of oral health as an integral component of general healthcare in later life. Given the high prevalence of edentulism in the screened population and the reduced oral health situation at admission, earlier intervention strategies are essential. Multiple approaches are necessary to engage patients and their families and to raise awareness of the need for assisted oral care.

## Figures and Tables

**Figure:**
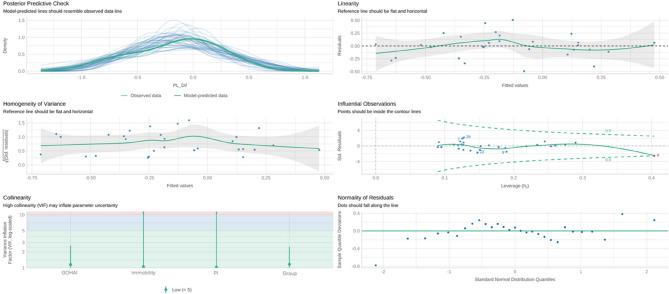
Appendix 2. Model diagnostics validating regression assumptions. Notes: The assumptions of normally distributed residuals and the absence of influential outliers were again verified through visual inspection and statistical tests, including Cook's distance and the Shapiro-Wilk test. Abbreviations: GOHAI, Geriatric Oral Health Assessment Index; Group, Intervention/Control Group; PI, Plaque Index at baseline

## Data Availability

The datasets used and/or analyzed during the current study are available from the corresponding author on reasonable request.

## Appendix

**Table:** Appendix 1. model selection procedure

Model-Selection	M1	M2	M3	M4
Model Intercept	**0.787***	**2.027*****	**1.776*****	**1.800****
*95% CI*	*(0.15;1.41)*	*(0.98; 3.08)*	*(0.80;2.75)*	*(0.77;2.8)*
Plaque Index, BL	−0.146	−0.140	−0.171**†**	−0.172**†**
*95% CI*	*(−0.38;−0.08)*	*(−0.35;0.07)*	*(−0.36;0.02)*	*(−0.36;0.02)*
Intervention	**−0.411****	**−0.516*****	**−0.475*****	-**0.480*****
*95% CI*	*(−0.65;−0.16)*	*(−0.74;−0.29)*	*(−0.69;−0.26)*	*(−0.70;−0.26)*
GOHAI		**>−0.023****	**−0.020***	**−0.020***
*95% CI*		*(−0.04;−0.01)*	*(−0.04;−0.01)*	*(−0.04;−0-01)*
Immobility			**0.245***	**0.249***
*95% CI*			*(0.05;0.44)*	*(0.04;0.46)*
Number of teeth				−0.001
*95% CI*				*(−0.02;0.01)*
Model Diagnostics				
Numbers Observed	29	29	29	29
R2	0.366	0.524	0.626	0.626
R2 Adj.	0.318	0.467	0.563	0.545
AIC	20.0	13.7	8.7	10.7
BIC	25.4	20.5	16.9	20.2
RMSE	0.30	0.26	0.23	0.23

*Abbreviations: AIC* Akaike Information Criterion, *BIC*, Bayesian Information Criterion, *BL* Baseline, *CI* Confidence Interval, *GOHAI* Geriatric Oral Health Assessment Index, *RMSE* Root Mean Square Error

*p-value < 0.05 considered significant (bold)

† *p* < 0.1

* *p* < 0.05

** *p* < 0.01

*** *p* < 0.001

† p-value < 0.1 > 0.05

## Abbreviations

BI: Barthel Index
CGA: Comprehensive Geriatric Assessment
CIRS: Cumulative Illness Rating Score
CPITN: Community Periodontal Index for Treatment Needs
DMFT: Decayed, Missing or Filled Teeth Index
EGCR: Early Geriatric Complex Rehabilitation
FU-1: Follow-up 1
FU-2: Follow-up 2
GOHAI: Geriatric Oral Health Assessment Index
MPI: Multidimensional Prognostic Index
PBI: Papillary Bleeding Index
PI: Plaque Index
PSI: Periodontal Screening Index
RCT: Randomised Controlled Trial
SoC: Standard of Care
